# Effects of Virtual Reality Intervention on Cognition and Motor Function in Older Adults With Mild Cognitive Impairment or Dementia: A Systematic Review and Meta-Analysis

**DOI:** 10.3389/fnagi.2021.586999

**Published:** 2021-05-05

**Authors:** Shizhe Zhu, Youxin Sui, Ying Shen, Yi Zhu, Nawab Ali, Chuan Guo, Tong Wang

**Affiliations:** ^1^Department of Rehabilitation, The First Affiliated Hospital of Nanjing Medical University, Nanjing, China; ^2^School of Rehabilitation Medicine, Nanjing Medical University, Nanjing, China

**Keywords:** meta-analysis, cognition, motor, virtual reality, mild cognitive impairment, dementia

## Abstract

**Background:** Virtual reality (VR) intervention is an innovative and efficient rehabilitative tool for patients affected by stroke, Parkinson's disease, and other neurological disorders. This meta-analysis aims to evaluate the effects of VR intervention on cognition and motor function in older adults with mild cognitive impairment or dementia.

**Methods:** Seven databases were systematically searched for relevant articles published from inception to April 2020. Randomized controlled trials examining VR intervention in adults with mild cognitive impairment or dementia aged >60 years were included. The primary outcome of the study was cognitive function, including overall cognition, global cognition, attention, executive function, memory, and visuospatial ability. The secondary outcome was motor function, consisting of overall motor function, balance, and gait. A subgroup analysis was also performed based on study characteristics to identify the potential factors for heterogeneity.

**Results:** Eleven studies including 359 participants were included for final analysis. Primary analysis showed a significant moderate positive effect size (ES) of VR on overall cognition (*g* = 0.45; 95% confidence interval (CI) = 0.31–0.59; *P* < 0.001), attention/execution (*g* = 0.49; 95% CI = 0.26–0.72; *P* < 0.001), memory (*g* = 0.57; 95% CI = 0.29–0.85; *P* < 0.001), and global cognition (*g* = 0.32; 95% CI = 0.06–0.58; *P* = 0.02). Secondary analysis showed a significant small positive ES on overall motor function (*g* = 0.28; 95% CI = 0.05–0.51; *P* = 0.018). The ES on balance (*g* = 0.43; 95% CI = 0.06–0.80; *P* = 0.02) was significant and moderate. The ES on visuospatial ability and gait was not significant. In the subgroup analysis, heterogeneity was detected in type of immersion and population diagnosis.

**Conclusions:** VR intervention is a beneficial non-pharmacological approach to improve cognitive and motor function in older adults with mild cognitive impairment or dementia, especially in attention/execution, memory, global cognition, and balance. VR intervention does not show superiority on visuospatial ability and gait performance.

## Introduction

Dementia is a collective name for a heterogeneous group of chronic neurodegenerative diseases characterized by progressive deterioration of goal-directed behaviors and cognitive function (Aruanno and Garzotto, 2019; D'Cunha et al., 2019). As the “symptomatic predementia stage” (Langa and Levine, 2014), individuals with mild cognitive impairment (MCI) display mild impairment in cognitive function with preserved independent functional abilities and with no obvious deficits in social and occupational functioning (Albert et al., 2011). However, these individuals typically have a higher risk of dementia than age-matched individuals without MCI (Petersen et al., 2018; Aruanno and Garzotto, 2019). According to the World Health Organization, ~47 million people worldwide are afflicted by dementia; this number is expected to increase to 75 million by 2030 and nearly 131 million by 2050 (Arvanitakis et al., 2019). In people aged 60 years and older, the reported prevalence of MCI ranges from 6.7 to 25.2% (Petersen et al., 2018).

Important breakthroughs in the pharmacological treatment of dementia have not been achieved, resulting in gravitation toward non-pharmacological approaches (D'Cunha et al., 2018). Updated practice guidelines have noted that exercises (Level B) and cognitive interventions (Level C) may be beneficial to improve measures of cognitive function in patients with MCI (Petersen et al., 2018) and stress that exercise training for 6 months is likely to improve cognitive outcome (moderate confidence in the evidence based on two Class II studies).

Virtual reality (VR) is a new technology for implementing rehabilitation of cognitive and motor function (Tieri et al., 2018). VR technology is defined as a “high-end computer interface that involves real time simulation and interactions through multiple sensory channels.” All VR applications possess two key features, immersion and interaction, which means that VR can bring the subject inside a virtual environment and to respond in real time to movements of the body in a naturalistic way (Tieri et al., 2018). VR can be categorized into three types according to the level of immersion: low immersion, semi-immersion, and full immersion (García-Betances et al., 2015). In a low immersive system, the patient interacts with the virtual environment using conventional graphic workstations such as PC monitor, keyboard and mouse. A semi-immersive VR system typically consists of more complicated interactive devices such as motion tracker, haptic gloves, and balance platform. On the other hand, a full immersive VR can be defined as an immersive experience delivered through a combination of more sophisticated graphic systems for example head-mounted display, surrounded screen, along with input of other sensory information such as sound, touch or even smell to let participants fully sink into virtual environment. VR intervention can combine exercises and cognitive training together making it a good option for patients with MCI or dementia.

According to recent meta-analyses, VR has significant beneficial effects on cognitive function in individuals who have sustained a stroke and evidence supports its use as an adjunct strategy for stroke rehabilitation at different stages of recovery using numerous platforms and training parameters (Aminov et al., 2018). VR can not only achieve the same effect as conventional rehabilitation training, but also improve gait and balance performance in patients with Parkinson's disease (Lei et al., 2019; Santos et al., 2019). To the best of our knowledge, there is only one systematic review regarding VR intervention in MCI or dementia (Kim et al., 2019). However, through analyzing the papers from Kim et al. (2019), we found that the quality of the included studies varied according to the Cochrane Collaboration's tool and the PEDro scale.

Due to the inclusion of many non-randomized controlled trials (RCTs) in Kim et al. (2019), the included articles are very different in study design, setting of control groups, and the quality of studies, all of which may increase the risk of bias. In this case, they only performed a sub-analysis of randomized vs. non-randomized studies. We believed this is not enough although it is difficult to analyze all potential risk of bias. Kim et al. (2019) only performed an overall effect size on cognition and physical fitness. Therefore, we perform an updated quantitative review of RCTs focusing on the efficacy of VR intervention on specific domains of cognition in older adults with MCI or dementia. In addition, knowing that MCI and dementia patients often have an increased risk of fall (Allali and Verghese, 2017), we also identify changes in motor function such as gait and balance.

The primary objective of this meta-analysis was to assess the effect of VR intervention on cognitive function, including overall cognition, global cognition, attention, executive function, memory, and visuospatial ability. The secondary objective was to identify the effect of VR on motor function, which comprises balance and gait.

## Materials and Methods

The results of this meta-analysis are reported in accordance with the Preferred Reporting Items for Systematic Reviews and Meta-Analysis (PRISMA) guidelines (Moher et al., 2009).

### Search Strategy

We searched the Cochrane Library, EMBASE, EBSCO, Ovid, PubMed, Scopus, and Web of Science databases from inception to April 2020 for RCTs that examined the effects of VR on one or more cognitive or behavioral outcomes in older adults with MCI or dementia without time limit. The specific search syntax, such as PubMed, can be available in the Supplementary Material.

### Eligibility Criteria

Eligibility criteria were formulated based on the PICOS framework (Moher et al., 2009):

- Participants: The age of the participants was >60 years, with a diagnosis of MCI or dementia (of any etiology).
- Intervention: We included studies using VR interventions that met the following definition: “The VR intervention should be the use of interactive simulations created with computer hardware and software to present users with a virtual figure to engage in environments that appear and feel similar to real world objects and events.” We did not limit the type of VR according to level of immersion (low immersive, semi-immersive, or full immersive). Interventions needed to be implemented for ≥5 h using standardized computerized tasks or interactive video games.
- Control: The comparison group received either an alternative intervention or no intervention. Alternative interventions included any activity designed to be therapeutic at the impairment, activity, or participation level that did not include the use of VR, ranging from motor and/or cognitive training to health education.
- Outcome: The primary outcome of this study is cognitive function, including overall cognition, global cognition, attention, executive function, memory, and visuospatial ability. The secondary outcome is motor function, consisting of balance and gait. All outcome measures had to be evaluated at baseline and immediately after the intervention period. Long-term follow-up data was not included in data synthesis, as only a small number of studies reported this information.
- Study: Eligible studies were peer-reviewed articles reporting results from RCTs that examined the effects of VR on one or more cognitive outcomes and motor function in adults aged ≥60 years with MCI or dementia. The following types of articles were excluded: (1) prospective or retrospective cohort studies; (2) case reports; (3) conference abstracts; and (4) not written in English.

### Study Selection

Two reviewers (SZ and YS) conducted the initial online search independently to avoid selection bias. After excluding duplicate studies, article titles, and abstracts were reviewed. If an abstract was considered relevant or ambiguous, the full text was reviewed and inclusion and exclusion criteria were applied. Disagreements regarding study eligibility were resolved by CG, who approved the final list of included studies.

### Data Extraction and Analysis

Data regarding study characteristics (nation, year, type of study, sample size, mean age, intensity, number of sessions, session length, immersion type of VR, comparison group, and outcome measures) of each article were extracted. We also summarized the components of the intervention in the two groups and extracted differences between them based on the description of interventions and the purpose of trials which could contribute to the superiority of VR. In the control group, a blank control group was defined as passive interventions while the same total training time in the experimental group was defined as active interventions. Most data was entered as mean with standard deviation (SD) for the VR and control groups at baseline and immediately after training. When mean and SD were not available, mean changes from baseline with SD or mean differences with 95% confidence interval (CI) were extracted. In addition, we contacted the authors to request raw summary data if there was no data available. For studies that included several tests of the same domain, each domain was averaged to one pooled effect size (ES) using Comprehensive Meta-Analysis (CMA) software version 2.0 (Biostat, Inc., Englewood, NJ, USA). For example, if a study included several attention tests, then these tests were summarized to generate one pooled effect size (Li et al., 2011). CMA allows for each of these different study outcomes to be flexibly entered into the model. A random effects model was used to correct for variable effect sizes across the studies if these studies show heterogeneity in their intervention (e.g., intervention type, duration, outcome measures) (Rosenblad, 2009). We chose the Hedges' g of the ES to estimate the efficacy of VR intervention. Hedge's g estimates of <0.3 were considered as small, ≥0.3 and <0.6 as moderate, and ≥0.6 as large, respectively (Hill et al., 2017). The calculation equation (Newton et al., 1998) of hedges' g is shown as follows:

(1) $$\begin{array}{r} g_{i}=\left(\left(m_{1i}-m_{2i}\right)/s_{i}\right)\left(1-3/\left(4N_{i}-9\right)\right) \end{array}$$

The collected data were utilized to calculate a combined effect size (Hedges' g) and 95% CI of changes in outcome measures between the experimental group (EG) and control group (CG) from pre- to post-test. Estimate variance was scaled up based on an assumed inter-correlation between the tests of 0.7 (Lampit et al., 2014; Hill et al., 2017). For Hedges' g, the direction of ES was positive if post-test performance was better than pre-test performance. In addition, a random effects model was chosen to accommodate heterogeneity for this analysis (Higgins et al., 2020). A subgroup analysis was also conducted based on study characteristics (population diagnosis, type of immersion, training time, and effectiveness of control group).

### Risk of Bias and Study Quality Assessment

The Cochrane Collaboration's tool (Higgins et al., 2011) was used to assess risk of bias in each individual study. The tool contains the domains of sequence generation; allocation concealment; blinding of participants, personnel, and outcome assessors; incomplete outcome data; selective outcome reporting; and other sources of bias. We classified items as “low risk,” “high risk,” or “unclear risk” of bias. The risk of bias is presented in Table 1.

**Table 1:** Study characteristics.

**Study**	**Country**	**Study design**	**Sample**				**VR intervention design**					**Additional therapy**	**PEDro score**
			**N (EG/CG)**	**Population diagnosis**	**Mean age**	**%Sex (Female)**	**Session Length/Per Week/N**	**Cost of time**	**Immersive type**	**VR content**	**Interactive medium**		
Delbroek et al. (2017)	Belgium	RCT	17 (8/9)	MCI	87.2	65	30 min/2/12	6 h	Semi	Task	BioRescue	No	8
Hughes et al. (2014)	America	RCT	20 (10/10)	MCI	77.35	70	90 min/1/24	36 h	Semi	Task	Wii/Motion tracking	No	7
Hwang and Lee (2017)	Korea	RCT	24 (12/12)	MCI	72.1	70.83	30 min/5/20	10 h	Semi	Task	Motion tracking	No	7
Liao et al. (2019)	China	RCT	34 (18/16)	MCI	74.37	67.64	60 min/3/36	36 h	Full	Task	HMD/stick/ Kinect	No	9
Man et al. (2012)	China	RCT	44 (20/24)	MCI	80.29	88.64	30 min/2or3/10	5 h	Low	Task	Joystick or a keyboard	No	7
Padala et al. (2012)	America	RCT	22 (11/11)	Mild AD	80.45	72.73	30 min/5/40	20 h	Semi	Task	Wii-Fit	Encourage self-training	7
Park et al. (2019)	Korea	RCT	21 (10/11)	MCI	72.04	80.95	30 min/3/18	9 h	Full	Task	HMD/depth camera/sensor motion tracker	No	8
Schwenk et al. (2016)	America	RCT	20 (11/9)	MCI	78.34	54.54	45 min/2/8	6 h	Semi	Task	Inertial sensors	No	8
Serino et al. (2017)	Italy	RCT	20 (10/10)	AD	87.65	85	20 min/3/10	200 min	Semi	Task	Gamepad	No	7
Tarnanas et al. (2014)	Greece	RCT	71 (32/39)	MCI	70.06	60.56	90 min/2/40	60 h	Semi	Task	Touching screen	No	9
Thapa et al. (2020)	Korea	RCT	66 (33/33)	MCI	72.65	76.47	100 min/3/24	40 h	Full	Task	HMD/headset/sticks	Health education	8

*EG, experimental group; CG, control group; N, number; AD, Alzheimer's disease; MCI, mild cognitive impairment; VR, virtual reality; RCT, randomized controlled trial; HMD, Head Mount Display*.

We also used the PEDro scale (Physiotherapy Evidence Database Rating Scale) to assess the quality of included studies using a score (Table 1) (Maher et al., 2003). The scale includes 11 items to rate study quality (Maher et al., 2003); the maximum score is 11. Studies that scored seven or higher were considered high quality, while those that scored six or lower were considered low quality. The scoring process was conducted by two authors (SZ and YS). CG established consensus scores and resolved any disagreements.

Publication bias was assessed by a funnel plot that displayed the relationship between sample size and ES. Small sample studies with a relatively large variance scatter at the bottom and large sample studies appear toward the top clustering around the mean ES. Studies that fall outside the funnel shape have a high risk of bias (Rosenblad, 2009).

## Results

### Identification of Studies

**Figure 1:**
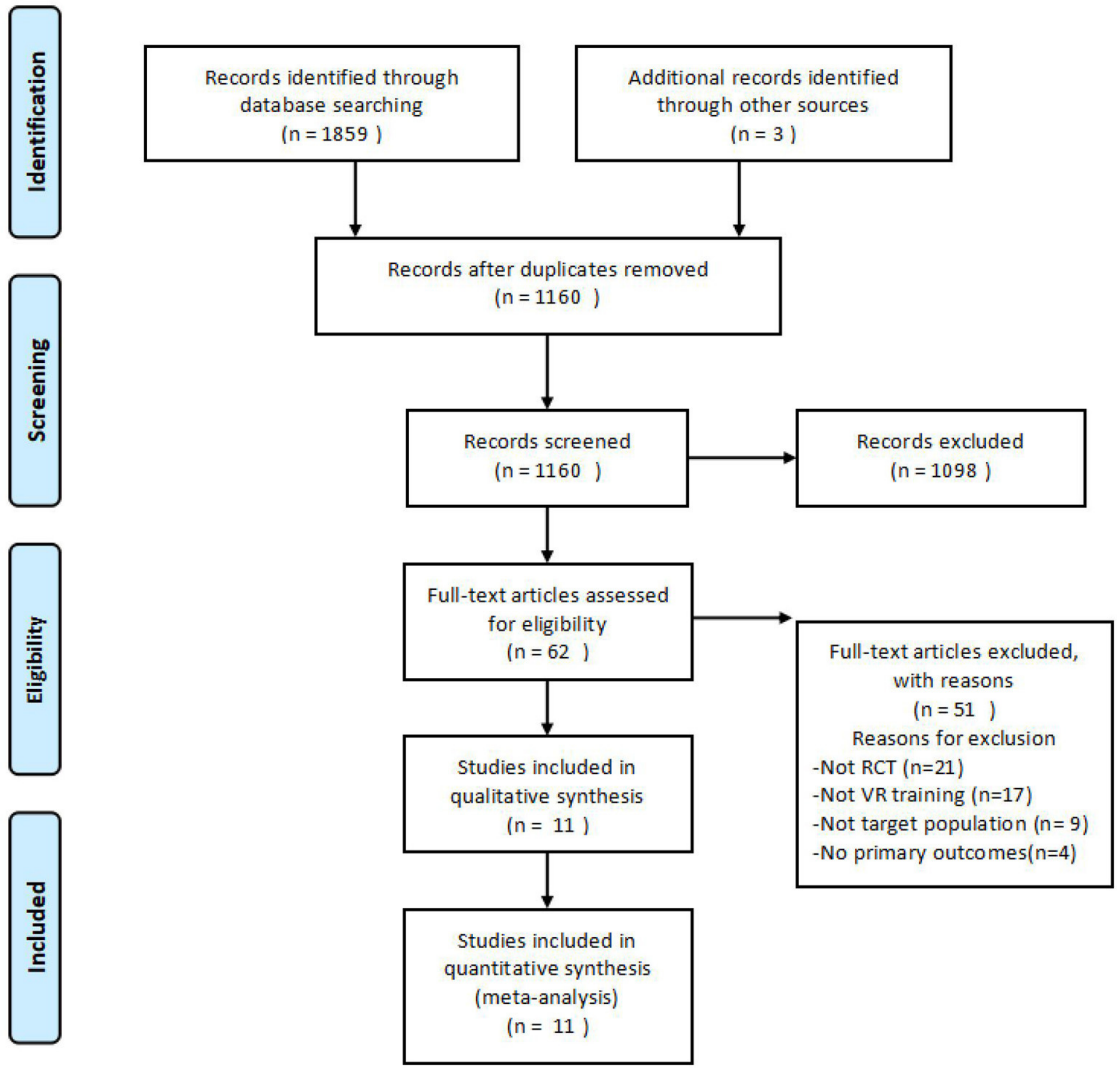
Flow chart of the literature search.

Figure 1 shows the flow diagram of study selection. The initial search yielded 1,859 articles from seven databases and three additional articles were identified through other sources. After removing duplicates, 1,160 eligible records were retrieved. After reading titles and abstracts, 1,098 articles were excluded. After readinging full text, the remaining 62 articles met the inclusion criteria. Among these 62 articles, 21 were excluded because study design was not RCT, 17 because VR was not used as the intervention, nine because participants did not meet the inclusion criteria, and four were excluded because exact outcome values were not reported and we contacted the authors, but there was no response. Finally, 11 original research articles were selected for further analysis.

### Participant and Study Characteristics

Table 1 summarizes the characteristics of the included studies. All 11 studies were RCTs. Nine studies reported older adults with MCI (*n* = 317) and two reported Alzheimer's dementia (*n* = 42). Thus, the analysis involved a total of 359 participants (experimental group = 175, mean group size = 16; control group = 184, mean group size = 17). Mean patient age was 75.84 years and 71.82% of the patients were female. Three studies reported full immersive VR, seven reported semi-immersive VR, and the remaining one reported low immersive VR. The VR methodology in all studies was task-oriented training. The interactive devices included head-mounted device, stick, Kinect, depth camera, sensor motion tracker, Nintendo Wii, touchscreen, keyboard, Bio Rescue, or gamepad. Number of intervention sessions ranged from 10 to 40 sessions. Training frequency varied from 1 to 5 sessions per week and the duration per session varied from 20 to 90 min. Three interventions in the control group were passive and eight interventions were active. The brief components of interventions in the two groups are listed in Table 2.

**Table 2:** Comparison of interventions.

**Study**	**VR group**	**Control group**	**Effectiveness of control group**	**Between-group difference**
Delbroek et al. (2017)	The BioRescue: containing the nine exercises which were used to train balance, weight bearing, memory, attention, and dual tasking, like walking through a street without touching barriers by performing weight shifts to the right and left side at a specific moment.	A blank control group.	Passive	Active training
Hughes et al. (2014)	Nintendo Wii group: including bowling, golf, tennis, and baseball.	Healthy aging education program:learning about and discussing age-specific health-related topics with professionals from the local academic and health care communities.	Active	Intensity and interest of training; The element of motor training; VE
Hwang and Lee (2017)	The virtual reality program: confirming a self-image and solving the problems presented through the screen, enhancing the motivation and active participation of users through various sensory feedback.	Traditional occupational therapy.	Active	Interest of training; Feedback system; VE
Liao et al. (2019)	Virtual reality-based physical and cognitive training: a simplified 24-form Yang-style Tai Chi, resistance exercise, aerobic exercise, and functional tasks in the forms of window cleaning, goldfish scooping and other tasks relevant to daily activities.	Combined physical and cognitive training:walking while reciting poems, naming flowers and animals while crossing obstacles, solving math questions during the resistance training, and so on.	Active	Different training items; Instant adjustment of difficulty; VE
Man et al. (2012)	VR programme: training in virtual environment of a home setting and a convenience shop, such as moving around, reading, and memorizing the items on a memo pad placed on the table within the living room.	Therapist-led programme:a sample memory training sheet showing the objects to be memorized and a sample answer sheet showing the objects to be memorized and distracters.	Active	Instant adjustment of difficulty; Variety in training; VE
Padala et al. (2012)	Wii-Fit group: including yoga, strength training, aerobics, and balance games.	Walking group:walking at their own pace as a group of three or four subjects at any given time with research personnel.	Active	ADL-oriented tasks; Intensity and interest of training; VE
Park et al. (2019)	MR-based cognitive training system: 15 training tasks that reflected daily activities where the study participants are likely to participate in a home setting, such as caring for a grandchild.	Computer-assisted training system:providing 10 training activities (two visual processing tasks that assess response time during visual stimulation, two auditory processing tasks that assess response time during auditory stimulation and so on).	Active	ADL-oriented tasks; Instant adjustment of difficulty; VE
Schwenk et al. (2016)	Exercise Training Technology: including ankle point-to-point reaching task (requiring anterior, posterior, and lateral leaning and partial weight transfer in order to improve postural balance during standing) and virtual obstacle-crossing task (crossing virtual obstacles moving on the computer screen from the left to the right side).	A blank control group.	Passive	Active training
Serino et al. (2017)	VR-Based Training Program: asking participants to enter this virtual city starting from the center of the scene to discover one, two or three hidden objects.	Traditional cognitive rehabilitative activities:cards games, naming, fluency, and music listening.	Active	Instant adjustment of difficulty; Different training items; VE
Tarnanas et al. (2014)	Virtual reality museum cognitive exercises: containing three tasks encompassing several tasks from each cognitive domain (memory, attention, execution), such as following instructions to locate and find items in an order and so on.	Learning-based memory training:Including viewing DVD-based educational programs on history, art and literature or participated at puzzle solving exercises.	Active	Reward-based daily training; VE
Thapa et al. (2020)	The VR training: consisting of four series of cognitive games (juice making, crow shooting, finding the fireworks number, memory object at the house).	A blank control group.	Passive	Active training

*VR, virtual reality; VE, virtual environment; ADL, activity of daily living*.

Table 3 provides an overview of the outcome measures used in the different studies.

**Table 3:** Outcome measures used in the included studies.

**Article**	**Attention/Execution**	**Memory**	**Visuospacial ability**	**Global cognition**	**Gait**	**Balance**
Delbroek et al. (2017)				MoCA	Instrument timed up and go	TT
Hughes et al. (2014)	TMT			The computerized assessment of mild cognitive impairment, Cognitive Self-report questionnaire	Gait speed	
Hwang and Lee (2017)	Word color test	Visual span test				Balance limit of stability
Liao et al. (2019)	TMT, SWCT				Gait system	
Man et al. (2012)		Fuld object memory evaluation, Multifactorial memory questionnaire				
Padala et al. (2012)				MMSE		Berg balance scale, TT
Park et al. (2019)	TMT, Verbal fluency test	Korean-BNT, word list test, Constructional recall	Constructional praxis			
Schwenk et al. (2016)	TMT			MoCA	Gait speed, Stride time	Center of mass
Serino et al. (2017)	Verbal fluency test, Verbal categorical test, Frontal assessment battery, Attentional matrices test	Digit span test, Corsi block test				
Tarnanas et al. (2014)	SWCT, Letter fluency test, TMT, Category fluency test	Rey auditory verbal learning test, BNT, Digit span test	Rey complex figure copy	MMSE		
Thapa et al. (2020)	TMT, Symbol digit substitution test			MMSE	Gait speed, 8-feet up and go	

*MoCA, Montreal Cognitive Assessment; TT, Tinetti Test; TMT, Trail Making Test; SWCT, Stroop Color and Word Test; BNT, Boston Naming Test; MMSE, Mini-Mental State Examination*.

(1) Attention/Executive Function: Trail Making Test (Arnett and Labovitz, 1995), Stroop Color and Word Test (Scarpina and Tagini, 2017), verbal fluency test (Park et al., 2019), verbal categorical test (Serino et al., 2017), Frontal Assessment Battery (Appollonio et al., 2005), Attentional Matrices Test (Serino et al., 2017), letter fluency test (Tarnanas et al., 2014), category fluency test (Tarnanas et al., 2014), and digit symbol substitution test (Makizako et al., 2013).

(2) Memory: visual span test (Hwang and Lee, 2017), Fuld Object Memory Evaluation (Monaco et al., 2015), Multifactorial Memory Questionnaire (Troyer and Rich, 2002), Boston Naming Test (Erdodi et al., 2018), word list test (Lee et al., 2002), constructional recall (Lee et al., 2004), digit span test (Monaco et al., 2013), Corsi block test (Serino et al., 2017), and Rey Auditory Verbal Learning Test (Lezak, 1998).

(3) Visuospatial ability: constructional praxis (Park et al., 2019) and Rey complex figure copy (Shin et al., 2006).

(4) Global Cognition: Montreal Cognitive Assessment (Nasreddine et al., 2005), Mini-Mental State Examination (Folstein et al., 1975), The Computerized Assessment of MCI (Saxton et al., 2009), and Cognitive Self-Report Questionnaire (Hughes et al., 2014).

(5) Gait: Gait speed, stride time, gait system (Liao et al., 2019), Instrument Timed Up and Go (Podsiadlo and Richardson, 1991), and 8-feet Up and Go (Thapa et al., 2020).

(6) Balance: Tinetti test (Tinetti et al., 1986), Balance Limit of Stability (Hwang and Lee, 2017), Berg Balance Scale (Muir et al., 2008), and center of mass (Pai and Patton, 1997).

### Primary and Secondary Analyses

**Figure 2:**
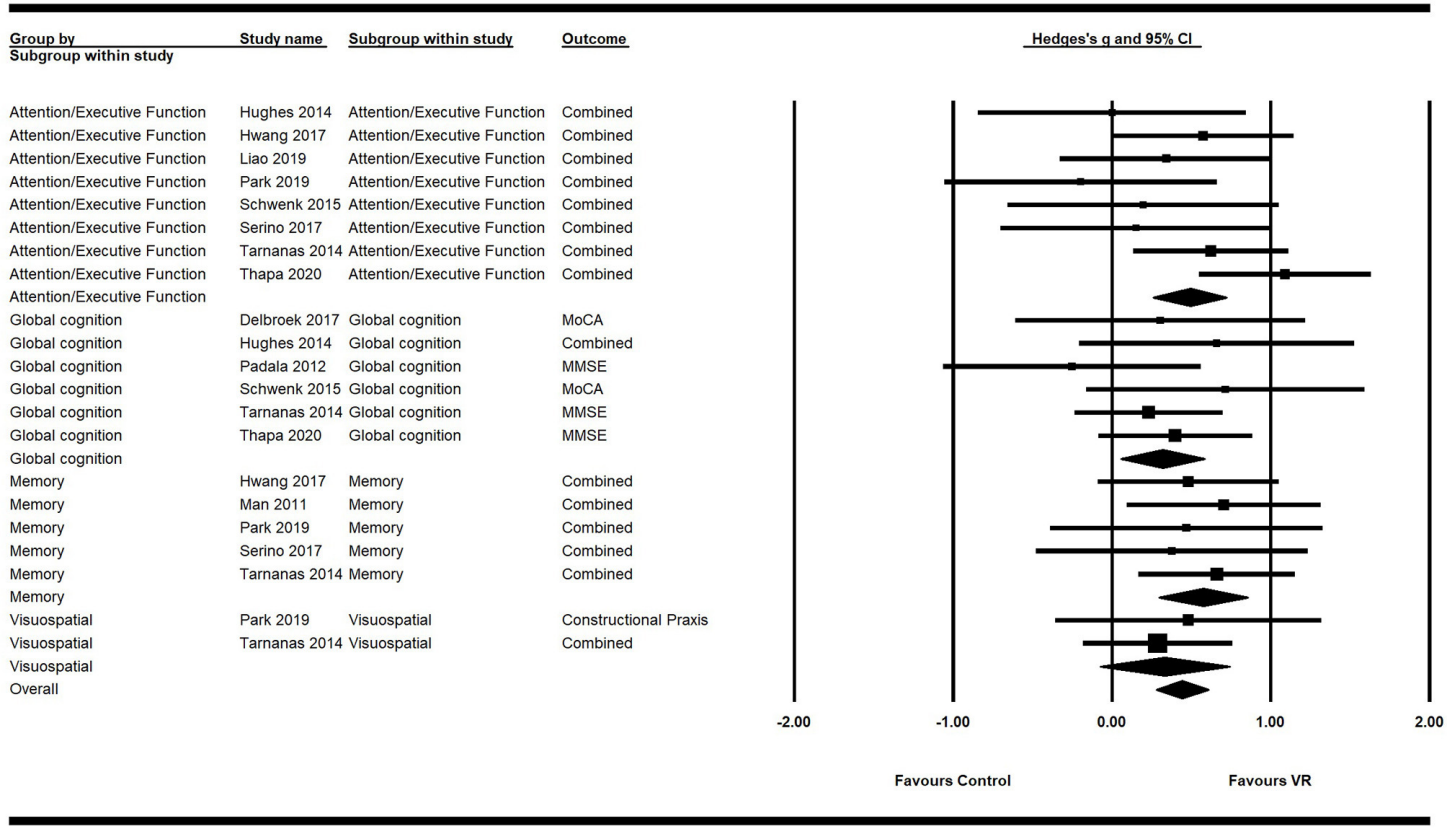
Forest plot for the efficacy of VR intervention on cognitive functions compared with the control group.

Primary analysis using the random effects model showed a significant moderate positive ES of VR on overall cognition (*g* = 0.45; 95% CI = 0.31–0.59; *P* < 0.001; *I*^2^ = 0%), attention/execution (*g* = 0.49; 95% CI = 0.26–0.72; *P* < 0.001; *I*^2^ = 31.45%), memory (*g* = 0.57; 95% CI = 0.29–0.85;*P* < 0.001; *I*^2^ = 0%), and global cognition (**g** = 0.32; 95% CI = 0.06–0.58; *P* = 0.02; *I*^2^ = 0%) (Figure 2; Table 4). The ES of VR on visuospatial ability was not significant (*g* = 0.33; 95% CI = −0.08–0.74; *P* = 0.11; *I*^2^ = 0%).

**Table 4:** Mean weighted effect sizes, confidence interval, and heterogeneity for primary and secondary outcome measures.

		**K**	**N**	**Standard error**	**95% CI**	**Q**	**P(Q)**	***I*^**2**^**
Cognitive functions	Global cognition	6	216	0.14	0.06–0.58	3.54	0.62	0.00
	Execution/Attention	8	300	0.12	0.26–0.72	10.21	0.18	31.45
	Memory	5	204	0.14	0.29–0.85	0.66	0.96	0.00
	Visuospatial ability	2	92	0.21	−0.08–0.74	0.16	0.69	0.00
	Overall cognition	11	359	0.07	0.31–0.59	16.71	0.67	0.00
Motor functions	Gait	6	179	0.15	−0.11–0.47	4.05	0.54	0.00
	Balance	4	107	0.19	0.06–0.80	1.37	0.71	0.00
	Overall motor function	7	203	0.12	0.05–0.51	6.47	0.69	0.00

*k, number of studies; N, number of patients; CI, confidence interval; Q, within domain heterogeneity; p(Q), p-value for heterogeneity; I^2^, percentage of heterogeneity due to true differences within studies, p < 0*.

**Figure 3:**
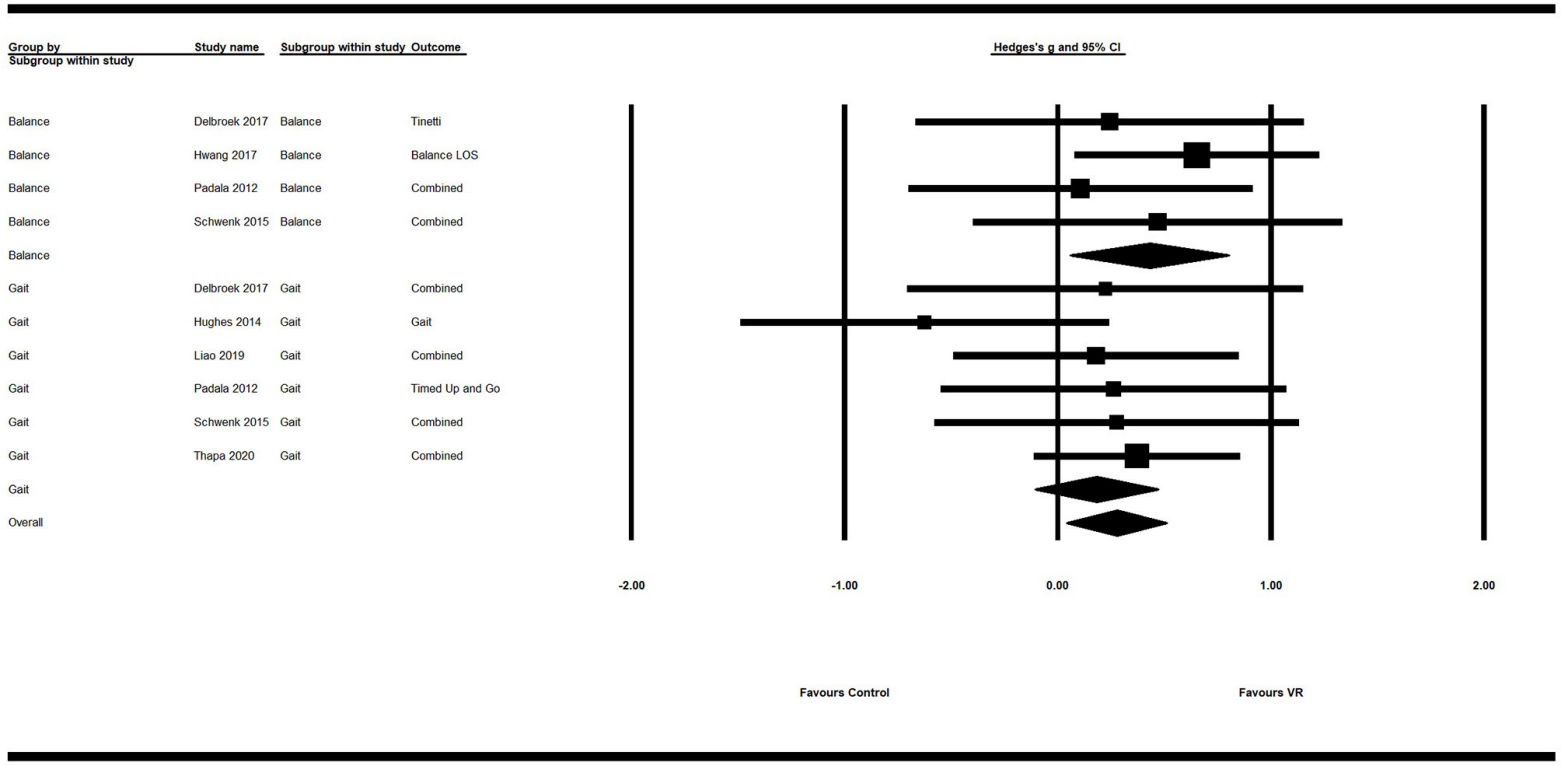
Forest plot for the efficacy of VR intervention on motor functions compared with the control group.

Secondary analysis showed a significant small positive ES of VR on overall motor function (*g* = 0.28; 95% CI = 0.05–0.51; *P* = 0.018; *I*^2^ = 0%). Its ES on balance (*g* = 0.43; 95% CI = 0.06–0.80; *P* = 0.02; *I*^2^ = 0%) was significant and of moderate efficacy. However, the ES of VR on gait was not significant (*g* = 0.18; 95% CI = −0.11–0.47; *P* = 0.21; *I*^2^ = 0%) (Figure 3; Table 4).

### Subgroup Analyses

The results of a subgroup analysis according to study characteristics are shown in Table 5. VR interventions for patients with MCI resulted in greater efficacy (*g* = 0.46; 95% CI = 0.25–0.68; *P* < 0.0001) than for patients with dementia (*g* = 0.11; 95% CI = −0.47–0.70; *P* = 0.706). Regarding type of immersion, studies using full immersive VR (*g* = 0.47; 95% CI = 0.10–0.83; *P* = 0.012) had a greater efficacy than studies using semi-immersive VR (*g* = 0.38; 95% CI = 0.11–0.64; *P* = 0.005), but did not reach statistical significance on low immersive VR (*g* = 0.54; 95% CI = −0.06–1.15; *P* = 0.077). For the training time, more than 20 h showed moderate effect size (*g* = 0.43; 95% CI = 0.15–0.70; *P* = 0.002), which were almost equal to those <20 h (*g* = 0.42; 95% CI = 0.12–0.72; *P* = 0.006). In terms of the effectiveness of interventions in the control group, active (*g* = 0.40; 95% CI = 0.15–0.64; *P* = 0.001) and passive (*g* = 0.55; 95% CI = 0.15–0.95; *P* = 0.008) interventions both showed moderate effect sizes.

**Table 5:** Effect sizes of subgroup according to study characteristics.

	**Categories**	**k**	**ES (Hedges's g)**	**+95% CI**		**p**	**SE**
Population diagnosis	AD	2	0.11	−0.47	0.70	0.706	0.30
	MCI	9	0.46	0.25	0.68	<0.0001	0.11
Type of immersion	Full	3	0.47	0.10	0.83	0.012	0.19
	Semi	7	0.38	0.11	0.64	0.005	0.14
	Low	1	0.54	−0.06	1.15	0.077	0.31
Training time	≥20 h	5	0.43	0.15	0.70	0.002	0.14
	>20 h	6	0.42	0.12	0.72	0.006	0.15
Effectiveness	Active	8	0.40	0.15	0.64	0.001	0.12
	Passive	3	0.55	0.15	0.95	0.008	0.21

*k, number studies; CI, confidence interval; ES, effect size; SE, standard error*.

### Risk of Bias and Study Quality

**Figure 4:**
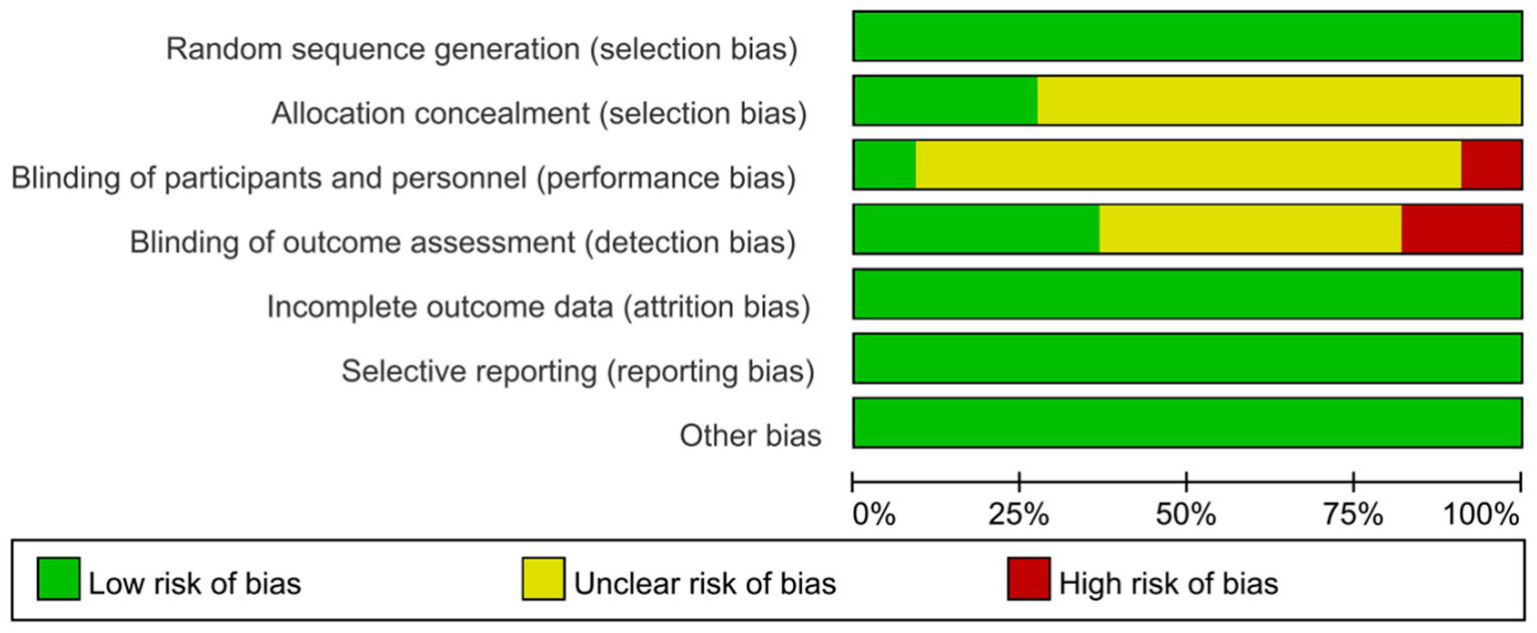
Risk of bias assessment per domain across studies with domains of bias on the Y-axis and % of studies having a high, unclear, or low risk of bias in each domain on the X-axis. The total score is the final author judgment of the total risk of bias.

Figure 4 and Table 6 show the risk of bias in the 11 included studies. All studies used random sequence generation (Man et al., 2012; Padala et al., 2012; Hughes et al., 2014; Tarnanas et al., 2014; Schwenk et al., 2016; Delbroek et al., 2017; Hwang and Lee, 2017; Serino et al., 2017; Liao et al., 2019; Park et al., 2019; Thapa et al., 2020). Participants and assessments were blinded in only one study (Padala et al., 2012). Four studies had adequate blinding of outcome assessment (Tarnanas et al., 2014; Delbroek et al., 2017; Liao et al., 2019; Park et al., 2019). All studies had a low risk of attrition bias on incomplete outcome data and selective reporting (Man et al., 2012; Padala et al., 2012; Hughes et al., 2014; Tarnanas et al., 2014; Schwenk et al., 2016; Delbroek et al., 2017; Hwang and Lee, 2017; Serino et al., 2017; Liao et al., 2019; Park et al., 2019; Thapa et al., 2020). Study quality is shown in Table 1. All 11 studies were high quality and the mean PEDro score was 7.67.

**Table 6:** Risk of bias assessment in included studies: the authors' judgments on each risk of bias item for all included studies.

**References**	**Sequence generation**	**Allocation concealment**	**Blinding**		**Incomplete outcome data**	**Selective outcome reporting**	**Other sources of bias**
			**Therapist and participants**	**Outcome assessors**			
Delbroek et al. (2017)	Low risk	Unclear	Unclear	Low risk	Low risk	Low risk	Low risk
Hughes et al. (2014)	Low risk	Unclear	Unclear	Unclear	Low risk	Low risk	Low risk
Hwang and Lee (2017)	Low risk	Unclear	High risk	Unclear	Low risk	Low risk	Low risk
Liao et al. (2019)	Low risk	Low risk	Unclear	Low risk	Low risk	Low risk	Low risk
Man et al. (2012)	Low risk	Unclear	Unclear	Unclear	Low risk	Low risk	Low risk
Padala et al. (2012)	Low risk	Unclear	Low risk	High risk	Low risk	Low risk	Low risk
Park et al. (2019)	Low risk	Unclear	Unclear	Low risk	Low risk	Low risk	Low risk
Schwenk et al. (2016)	Low risk	Low risk	Unclear	Unclear	Low risk	Low risk	Low risk
Serino et al. (2017)	Low risk	Unclear	Unclear	High risk	Low risk	Low risk	Low risk
Tarnanas et al. (2014)	Low risk	Unclear	Unclear	Low risk	Low risk	Low risk	Low risk
Thapa et al. (2020)	Low risk	Low risk	Unclear	Unclear	Low risk	Low risk	Low risk

**Figure 5:**
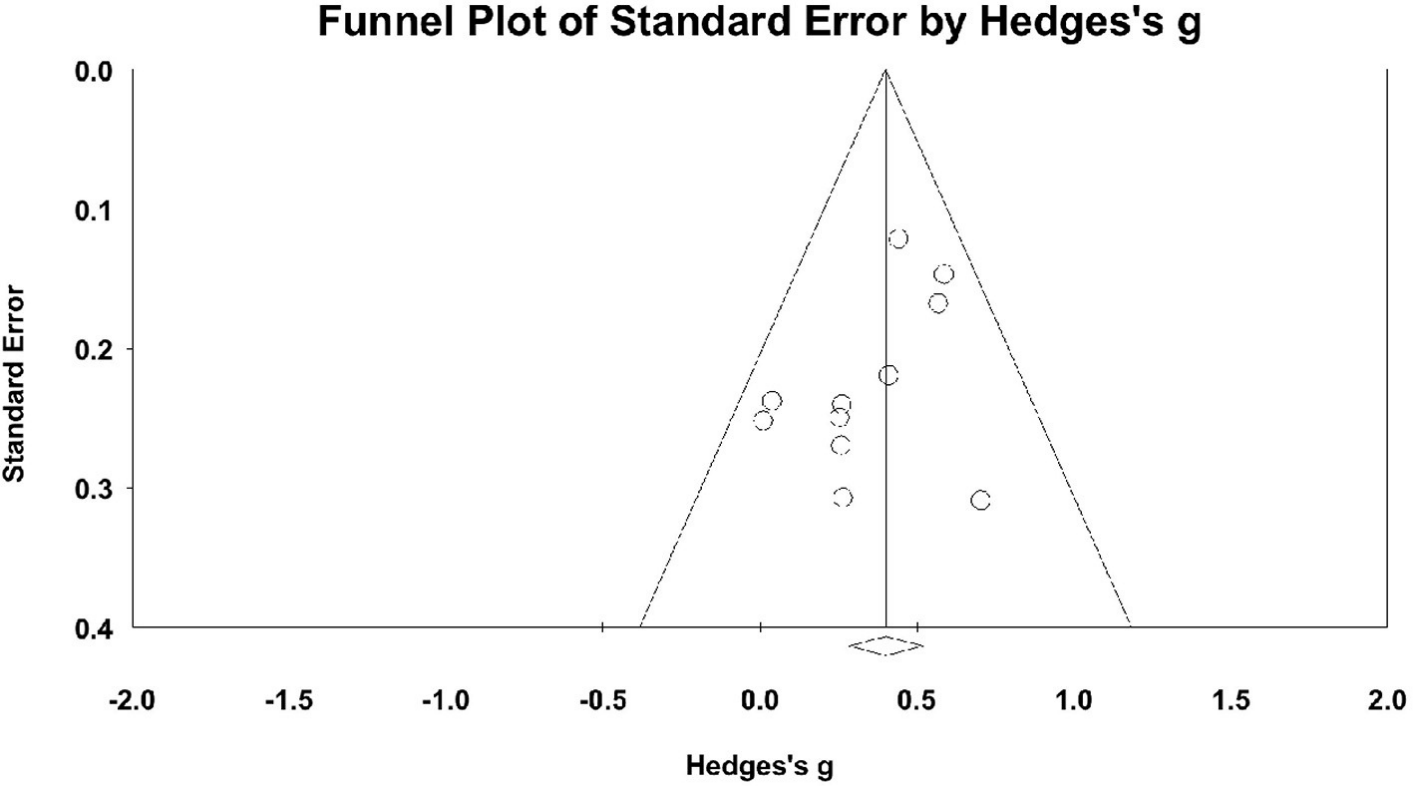
Funnel plot for overall cognition with Hedge's g on the X-axis and the standard error on the Y-axis.

Figure 5 shows the funnel plot of the studies. Distribution was fairly symmetric, indicating no hint of publication bias. Orwin's fail-safe N was calculated only for the measures that showed significant differences between the experimental and control groups. For overall cognition, 71 studies would be required to reduce the observed effect to an ES <0.1.

## Discussion

Based on the results from 11 high quality RCTs, this meta-analysis showed that VR intervention exhibited a moderate effect on several measures of cognitive and motor function, including attention/execution, memory, global cognition, and balance. This indicates VR is a promising effective non-pharmacological therapy for older adults with MCI or dementia. However, VR intervention did not show a significant effect on visuospatial ability or gait.

### Interpretation of Results and Comparison With Previous Research

To the best of our knowledge, this is the first meta-analysis to focus on the effect of VR intervention on specific cognitive and motor function domains in older adults with MCI or dementia. Recently, Kim et al. (2019) investigated the benefits of VR intervention in older adults with MCI or dementia. In their meta-analysis, four of the 11 included studies were not RCTs. However, by including only RCTs, our meta-analysis was able to quantify the magnitude of the effect, confirming the efficacy of VR intervention in MCI or dementia patients (Karssemeijer et al., 2017). Moreover, our exploration of the effects of VR intervention on specific cognitive and motor function domains can provide a framework for future clinical practice.

Our results are comparable with those of Kim et al. (Cohen' *d* = 0.42; 95% CI = 0.24–0.60), demonstrating a moderate effect of VR on cognitive function (Kim et al., 2019). However, they did not perform an analysis of specific cognitive domains and found that VR had no significant effect on execution (Cohen' *d* = 0.07; 95% CI = −0.34–0.49), in contrast to our findings. Another recent meta-analysis that evaluated the effect of combined cognitive and physical exercise training on cognitive function in older adults with MCI or dementia did not find a significant effect on the attention/execution cognitive domain (SMD = 0.38; 95% CI = −0.21–0.97) or memory (SMD = 0.02; 95% CI = −0.35–0.39) either (Karssemeijer et al., 2017), which is inconsistent with our findings. However, the combined ES data in their study were based on only two studies. Furthermore, whether VR intervention utilizes its unique features of immersion and interaction to improve these cognitive domains remains to be explored. A meta-analysis conducted by Hill et al. (2017) showed a positive effect of computerized cognitive training on working memory (Hedges' *g* = 0.74; 95% CI = 0.32–1.15), verbal memory (*g* = 0.42; 95% CI = 0.21–0.63), and attention (*g* = 0.44; 95% CI = 0.20–0.68) in older adults with MCI or dementia. They found no significant effect on visuospatial ability (*g* = 0.18; 95% CI = −0.23–0.60), which is consistent with our findings. Another recent review noted that VR was more effective in improving attention, visuospatial deficits, and motor impairment in stroke patients (Maggio et al., 2019). Of all the studies included in our meta-analysis, only two focused on MCI subjects and provided visuospatial ability data. Therefore, future studies are needed to determine if VR intervention can improve visuospatial ability in older adults with MCI or dementia. Meanwhile, tasks of VR interventions in these two studies were not especially designed for the visuospatial ability, so the effects could not be translated into the significant improvement of a general visuospatial ability compared with the interventions in the control group. Our meta-analysis showed a moderate ES of VR intervention on global cognition (SMD = 0.32; 95% CI = 0.17–0.47), which is in agreement with the results of Karssemeijer et al. (2017) and Hill et al. (2017).

Kim et al. (2019) also reported a moderate ES of VR intervention on overall motor function (Cohen' *d* = 0.41; 95% CI = 0.16–0.65), but did not evaluate the effect on measures of gait, balance, or falls. Balance improvement (MD = 2.99; 95% CI = 1.80–4.18) was in accordance with the efficacy of VR intervention in community-dwelling older adults found by Neri et al. (2017). Their study also showed no significant effect on gait performance (MD = −1.20; 95% CI = −1.62 to −0.77), which is consistent with our study. Indeed, in addition to cognitive impairment, patients with MCI or dementia likely have impaired motor function, which is often ignored in current studies. Poor performance in motor function is seldom seen in a single walking or motor task, since patients may sacrifice their efficiency in cognitive tasks to compensate for deficits in motor performance; however, this relationship is broken when a complex cognitive task is added to complete concurrently (Bloem et al., 2006). Therefore, in future studies, researchers should take dual tasking into consideration when designing measurements of motor outcomes.

In order to understand what makes the VR intervention efficient, we did a subgroup analysis according to study characteristics, as follows: (1) population diagnosis (e.g., MCI., AD.); (2) type of immersion (e.g., full immersion, semi-immersion, low immersion); (3) training time (e.g., less or more than 20 h); (4) effectiveness of control group (e.g., active or passive interventions). We have found positive effect of VR intervention in the MCI group while no statistical significance in AD. It may be related to the progression of MCI to dementia that MCI as the “predementia stage” is less severe compared to AD (Langa and Levine, 2014; Roberts et al., 2014). Consequently, we assumed that VR may be a beneficial intervention for patients with MCI and the intervention in the early stage of cognitive impairment is very important. With different types of immersion, a deeper sense of immersion can provide the users with more real experience. This might explain why full immersive and semi-immersive VR showed efficacy. Previous studies demonstrated that the more sophisticated VR technology used, in terms of the degree of immersion, their participants would get deeper experience of the virtual environment (Tieri et al., 2018). One thing we have noticed is that there is no statistical significance in low immersive VR type, however, it is highly accepted by the older adults, due to low “cibersikness” symptomatology (An and Park, 2018). The subgroup analysis did not show obvious differences based on training time. However, we believe that total training time may be a factor affecting the outcome. Therefore, more attention should be paid to training time in future studies. We found VR interventions had better effectiveness than traditional strategies in the control group, no matter whether the intervention in the control group was active or passive.

### Mechanism and Shortcomings of VR

The mechanism behind VR technology for patients with MCI or dementia is still unclear and few studies have tried to explore it. As mentioned before, VR intervention has two unique features, immersion and interaction, which are thought to be the mechanisms behind the beneficial effect of VR technology. The feature of immersion can bring the sense of embodiment (Tieri et al., 2018), just likes “Avatar” to simulate a virtual body that substitutes the real one is able to elicit illusory sensations that virtual body itself belongs to the observer (Slater et al., 2008). According to neuroscience, to regulate and control the body in the world effectively, the brain creates an embodied simulation of the body in the world used to represent and predict actions, concepts, and emotions (Barrett, 2017), and VR just shares the similar basic mechanism to the brain to bring the simulated body into the virtual environment. The feature of immersion can elicit real physiological and psychological reactions (Meehan et al., 2005; Miller et al., 2008). For example, Salter (Slater et al., 2008) showed electromyography activity on the right arm of the participants when they observed an embodied virtual limb rotating on itself. At the same time, exposure to an immersive virtual environment having a whole hole on the floor has been shown to be able to elicit a stressful state determined by an increase in heart rate (Meehan et al., 2005). In our subgroup analysis, we have found that low immersive VR has no statistical significance in patients with MCI or dementia, but semi- and full immersive VR have, indicating that immersion plays an important role in the improvement of function in these patients.

Another unique feature is interaction which plays an important role in the immersion, feeds back the information in real time and has a reward system. By combining different external equipment such as motion tracker, headphone, haptic gloves, we can deliver the information of changes in the real body, resulting from the reactions to virtual environment, to the virtual body and easily set up the association of the virtual body with the virtual environment (Slater, 1999). In addition, the feedback system can provide the participants with the instant information that can be used to reinforce control of movement parameters and to reduce compensation movements (Subramanian et al., 2013) from judging these details. Finally, the reward system can arise the motivation of the participants to facilitate the repetition of body movement as well as improve patient compliance, treatment endurance, and happiness (Burdea et al., 2015; De Luca et al., 2018). Schmidt et al. (2012) also found that neuroplasticity process in the ventral striatum by connecting the motor and cognitive circuits can be enhanced by rewards.

However, these two features of VR technology are not independent and they influence each other. Therefore, to avoid one and explore the mechanism of the other is difficult. The above mechanism is our speculation, so future trials should try to set up a reasonable comparable group to explore these features respectively and help clarify the underlying mechanism of VR.

Furthermore, among current trials of VR interventions for patients with MCI or dementia, there are still few limitations. First, all of 11 included studies are goal-orientated that could be difficult for patients with severe cognitive impairment, like dementia, to finish such tasks. Our subgroup analysis showing no statistical significance in dementia may be the result of training difficulty. The efficacy of VR may reduce because the majority of the VR devices used are commercialized and few have adjusted their components to fit the needs of these patients. Second, although VR as a non-invasive, non-pharmacological cognitive rehabilitation intervention has gained increasing attention in recent years, health and safety must be taken into consideration, especially when intended for the use in older adults with neurodegenerative diseases. Cyber-sickness, a visually induced motion sickness reaction (Nooij et al., 2018) that can arise during or after immersion with a virtual environment depending on the level of immersion, should be a matter of concern in clinical settings (Bohil et al., 2011). Third, the conditions of the intervention between the groups are not the same among the included trials. By comparing differences between VR and control groups, we found there were more than one difference among these trials such as instant adjustment of difficulty, feedback system, and virtual environment. The question arises that whether one of them, like the element of the task, will produce the same efficacy as VR or no. The best way to answer this question is to find articles where the VR group set up a task and the control group performed the same task without VR at the same time. However, there is still a lack of such articles. The goal-oriented training has been proved to be effective in the recovery of many neurological diseases and all of the included studies used it in their VR training. At the same time, an advantage of VR is to combine such elements into their training. In future trials, it would be better to design a protocol to explore a single variable between experimental and control groups which could help us to learn more about the efficacy of that specific variable.

### Strengths and Limitations

The strength of this meta-analysis is that only RCTs were included. Furthermore, to the best of our knowledge, this is the first meta-analysis to focus on both cognitive and motor functions as well as specific cognitive domains. However, several limitations should be addressed. First, the intervention characteristics varied between studies. The optimal frequency and duration of intervention remain to be explored to maximize intervention effects. Second, as the training sessions in each study are diverse, we only evaluated the immediate results after VR intervention to avoid bias. Therefore, the follow-up effect of VR in MCI or dementia was not analyzed so we could not estimate whether VR can prevent long-term worsening of dementia or conversion of MCI to dementia without persistent training. Third, due to the relatively small number of participants in the included studies, it is statistically inappropriate to analyze the impact of varied intervention components or different subtypes and severities of MCI or dementia.

### Implications for Future Studies

Further research is needed to explore the most effective characteristics of VR intervention, specifically examining type, frequency, and duration of intervention as well as immersion. In addition, large sample studies are needed and long-term effects should be studied to gain insight into possible maintenance effects. Finally, future studies should investigate the effects of VR intervention on changes in neuroimaging findings and molecular markers.

## Conclusion

Our meta-analysis shows that VR intervention is a beneficial non-pharmacological approach to improve cognitive and motor function in older adults with MCI or dementia, especially in attention/execution, memory, global cognition, and balance. Moreover, VR intervention does not show superiority on visuospatial ability and gait performance. The clinical relevance of our findings remains to be confirmed in future research.

## Data Availability Statement

The raw data supporting the conclusions of this article will be made available by the authors, without undue reservation.

## Author Contributions

SZ, YSu, YSh, and YZ chose the topic. SZ, YSu, and YSh performed the analysis. SZ, YSu, and YZ analyzed the data. CG, TW, and NA participated in the whole process. CG and TW made final decisions. All authors contributed to writing of this manuscript.

## Conflict of Interest

The authors declare that the research was conducted in the absence of any commercial or financial relationships that could be construed as a potential conflict of interest.
